# Central nesfatin-1-expressing neurons are sensitive to peripheral inflammatory stimulus

**DOI:** 10.1186/1742-2094-6-27

**Published:** 2009-09-24

**Authors:** Marion S Bonnet, Emilie Pecchi, Jérôme Trouslard, André Jean, Michel Dallaporta, Jean-Denis Troadec

**Affiliations:** 1 Centre de Recherche en Neurobiologie-Neurophysiologie de Marseille (CRN2M), UMR 6231 CNRS, Marseille, France; 2 Département de Physiologie Neurovégétative, USC INRA 2027, Université Paul Cézanne, Université de la Méditerranée, Marseille, France

## Abstract

Recently, a novel factor with anorexigenic properties was identified and called nesfatin-1. This protein (82 aac) is not only expressed in peripheral organs but it is also found in neurons located in specific structures including the hypothalamus and the brainstem, two sites strongly involved in food intake regulation. Here, we studied whether some of the neurons that become activated following an injection of an anorectic dose of lipopolysaccharides (LPS) exhibit a nesfatin-1 phenotype. To this end, we used double immunohistochemistry to target the expression of the immediate-early gene *c-fos *and of nesfatin-1 on coronal frozen sections of the rat brain. The number of c-Fos+/nesfatin-1+ neurons was evaluated in the immunosensitive structures reported to contain nesfatin-1 neurons; i.e. paraventricular hypothalamic nucleus (PVN), supraoptic nucleus (SON), arcuate nucleus (ARC) and nucleus of the solitary tract (NTS). LPS strongly increased the number of c-Fos+/nesfatin-1+ neurons in the PVN, SON and NTS, and to a lesser extent in the ARC. Triple labeling showed that a portion of the nesfatin-1 neurons activated in response to LPS within the NTS are catecholaminergic since they co-express tyrosine hydroxylase (TH). Our data therefore indicate that a portion of nesfatin-1 neurons of both the hypothalamus and brainstem are sensitive to peripheral inflammatory signals, and provide the first clues suggesting that centrally released nesfatin-1 may contribute to the neural mechanisms leading to endotoxaemic anorexia.

## Findings

During infection and inflammation, the immune, endocrine and nervous systems closely interact to coordinate a range of physiological and behavioral changes known as the acute phase reaction. The behavioral symptoms collectively referred to as "sickness behavior" include fever, anorexia, adipsia, lethargy and reduction in social interactions [1,2]. Cytokines peripherally released act on specific central nuclei involved in feeding and homeostatic regulation, which leads to the central component of the acute phase response. The complex neuronal circuitry involved in the coordinated response to inflammation, which includes notably the nucleus of the solitary tract (NTS), area postrema (AP), ventrolateral medulla, parabrachial nucleus, paraventricular hypothalamic nucleus (PVN), supraoptic nucleus (SON), arcuate nucleus (ARC) and central nucleus of the amygdala, has been identified mainly by quantification of the immediate-early gene *c-fos *[3,4]. The expression of the *c-fos *gene is widely considered to be a high resolution marker of neuronal activity, since a body of evidence demonstrates that Fos protein is expressed in neurons whose activity is strongly stimulated by synaptic input [5]. Used in combination with immunohistochemical determination of neuronal phenotype, this approach has led to the identification and location of neurons activated by inflammation and involved in the triggering of sickness behavior [6-8].

Recently, Oh-I and co-authors reported the identification of a novel factor with anorexigenic properties, which they called nesfatin-1 [9]. Nesfatin-1 is reported to reduce food intake both after central (3^rd ^ventricle) and peripheral administration [9,10]. Apart from adipose tissue, from which nesfatin-1 was first isolated, this protein is also expressed by gastric mucosa [11] and pancreatic β-cells [12]. This pattern of nesfatin-1 expression, as well as the presence of nesfatin-1 within the plasma of rodents and humans, strongly suggests that this compound may act as a circulating regulatory factor. Interestingly, expression of nesfatin-1 within the brain has been reported by various groups [13-16]. Neurons expressing nesfatin-1 are found located in various areas including the brainstem (NTS, dorsal motor nucleus of the vagus: DMNX) and hypothalamic nuclei (ARC, PVN, SON).

In the present study, we investigated, by mean of a c-Fos/nesfatin-1 double immunohistochemical approach, whether a portion of those neurons activated in response to peripheral injection of an anorexic dose of lipopolysaccharides (LPS) exhibit nesfatin-1 expression. The peripheral injection of LPS is a well characterized model of inflammation, known to induce events occurring during sepsis such as the synthesis and release of inflammatory cytokines and the activation of immunosensitive structures in the brain [2].

Adult male Wistar rats weighing 250-300 g (Janvier, France) were housed individually in a pathogen-free facility at controlled temperature on a 12:12-h light/dark cycle (lights off at 18.00 h) with food (AO4, SAFE UAR, France) and water available *ad libitum*. All procedures were in accordance with the European Directive N886/609 and the local committees for animal use and care. For habituation, animals were handled and injected intraperitoneally (i.p.) with physiological saline every day for at least seven days before experiment. On the day of the experiment, animals (n = 6 for each condition) were fasted at 9.00 h and the food was withdrawn until the animal sacrifice in order to avoid activation of nesfatin-1 neurons consecutive to feeding. Four hours after the beginning of fasting, animals were injected with either saline or an LPS-containing solution (0.25 mg/kg, from *E. coli*, serotype 0127:B8, Sigma). The injection volume for all animals was 0.01 ml per gram of body weight. The rats were sacrificed three hours after injection, which corresponds to the peak of neuronal c-Fos immunoreactivity in hypothalamus and brainstem nuclei following peripheral administration of endotoxin [17]. After being deeply anaesthetized with ketamine (100 mg/kg i.p.; Merial, France) and xylazine (16 mg/kg i.p.; Bayer, France), rats were transcardially perfused with freshly depolymerized 4% PFA. The brains were removed immediately, post-fixed one hour in 4% PFA at 4°C and then cryoprotected for 24 to 48 hours in 30% sucrose at 4°C.

To identify activated nesfatin-1 neurons, double immunohistochemistry for c-Fos and nesfatin-1 was performed on brain sections. After freezing the brains in isopentane (-40°C), coronal sections (40 μm thick) were cut on a cryostat (Leica CM3050, France) and collected serially in 12-well plates containing PBS (0.1 M). Brains were cut at the brainstem level (Interaural -5.3 mm to -3.8) and at the forebrain level (Interaural +4.7 to +7.7 mm) according to the rat brain atlas from Paxinos and Watson [18]. The free-floating sections were incubated in a solution containing 0.3% H_2_O_2 _in PBS 0.1 M for quenching of endogenous peroxidase activity. A 1 h incubation in PBS containing 3% normal goat serum (NGS) and 0.3% Triton X-100 was performed to block non-specific binding sites. Sections were then treated for 48 hours at 4°C in PBS containing 3% NGS, 0.3% Triton X-100 and anti-c-Fos rabbit antiserum synthesized against amino acids 4-17 of human protein (1:10000, Ab-5, Calbiochem). After three washes in PBS, the sections were incubated overnight at 4°C with the biotinylated goat anti-rabbit IgG (1:400, Vector Labs). After further washes, sections were incubated with avidin-biotin complex (1:200, ABC Vector Elite kit) for 2 h at room temperature. Horseradish peroxydase activity was then visualized using a nickel-enhanced diaminobenzidine (DAB) as the chromogen to reveal a purplish-black stain of c-Fos-immunoreactive nuclei. The reaction medium contained nickelous ammonium sulphate (0.6%), DAB (0.006%) and H_2_O_2 _(0.02%) in PBS. The reaction was closely monitored and terminated when optimum intensity was achieved (10 min) by washing the sections in distilled water. Non-specific labeling was assessed on alternate slides that were treated identically to the above but in which the primary antibody was omitted. Sections were then incubated overnight at 4°C with a primary antibody raised against nesfatin-1 (1:10000, H-003-22-B, Phoenix Pharmaceuticals Inc.), washed in PBS, and incubated for 2 h at room temperature with a secondary antibody conjugated with Alexa 488 (1:400, Vector Labs). An additional labeling of the tyrosine hydroxylase (TH) enzyme was performed on brainstem sections. For this purpose, sections were incubated overnight at 4°C with anti-TH antibody (1:1000, MAB318, Chemicon), and revelation was performed using an Alexa 594-conjugated secondary antibody (1/400, Vector Labs). Finally, all sections were mounted on gelatin-coated slides, air dried, and coverslipped with mounting medium for fluorescence microscope preparation (DAKO).

For the analysis of neuronal activation, images of brain sections were acquired using 10× and 20× lenses with a DXM 1200 Camera (Nikon) coupled to ACT-1 software. Fluorescent images acquired at 520 nm were merged with phase contrast images to allow visualization of c-Fos+/nesfatin-1+ neurons. For each brain structure studied, the counts performed on four distinct sections from both hemispheres were averaged to yield the number of c-Fos nuclei and c-Fos+/nesfatin-1+ cells per section. Statistical analysis of data was performed with Sigmastat (version 3.5; Systat software Inc) using Student's *t*-test. P values less than 0.05 were considered significant.

**Figure 1:**
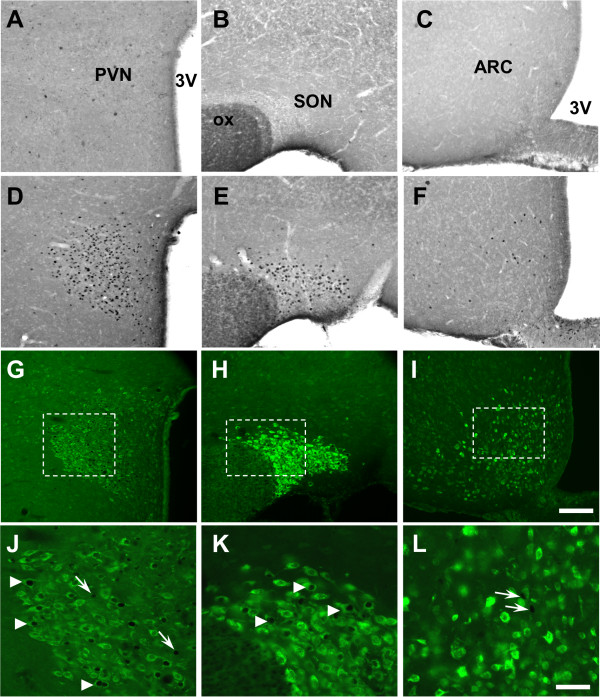
**A-F: Representative photomicrographs of c-Fos immunoreactivity in coronal sections of the PVN, ARC and SON**. Images derived from saline-injected (**A-C**) and LPS-treated (**D-F**) rats. **G-L**: Analysis of c-Fos and nesfatin-1 (green) double-labeling in coronal sections through the hypothalamus of LPS-treated rats. Dashed boxes in low magnification images (**G-I**) indicate the area where high magnification images (**J-L**) originate. Arrowheads: c-Fos+/Nesf+ neurons; arrows: c-Fos+ neurons negative for nesfatin-1. ARC arcuate nucleus; ox, optic chiasm; PVN, paraventricular nucleus of the hypothalamus; SON: supraoptic nucleus; 3V, third ventricle. Scale bars: 200 μm (A-I), 50 μm (J-L).

**Figure 2:**
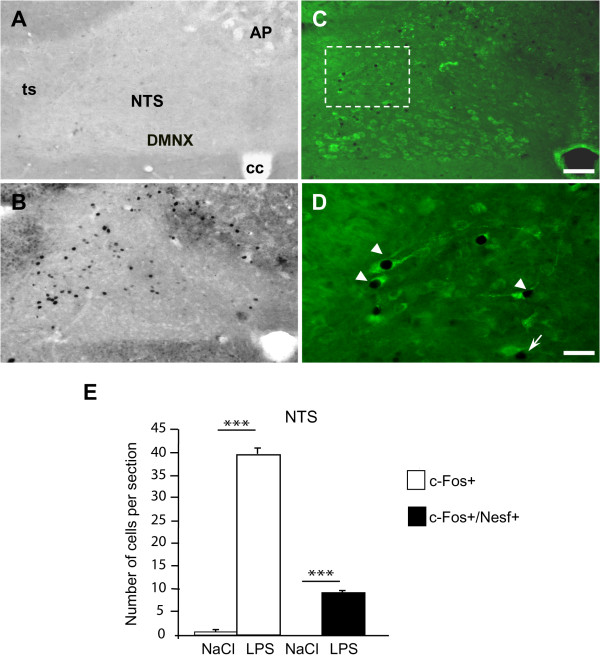
**A-B: c-Fos immunoreactivity within the DVC**. Representative coronal sections of the DVC of saline-injected (**A**) and LPS-treated (**B**) rats. **C-D**: c-Fos and nesfatin-1 (green) double-labeling in coronal sections through the DVC of LPS-treated rats. **D**: High magnification of c-Fos/nesfatin-1 positive neurons in the NTS. Dashed box in C indicates the area where high magnification image originates. Arrowheads: c-Fos+/Nesf+ neurons; arrow: c-Fos+ neuron negative for nesfatin-1. **E**: Quantification of the number of c-Fos and c-Fos/nesfatin-1 immunoreactive cells at the NTS subpostremal level of rats injected with either saline or LPS solution. Starred values are significantly different from saline-injected animals, *** p < 0.001. AP, area postrema; cc, central canal; DMNX, dorsal motor nucleus of the vagus; NTS, nucleus of the solitary tract; ts, tractus solitarius. Scale bars: 100 μm (A-C), 50 μm (D).

**Figure 3:**
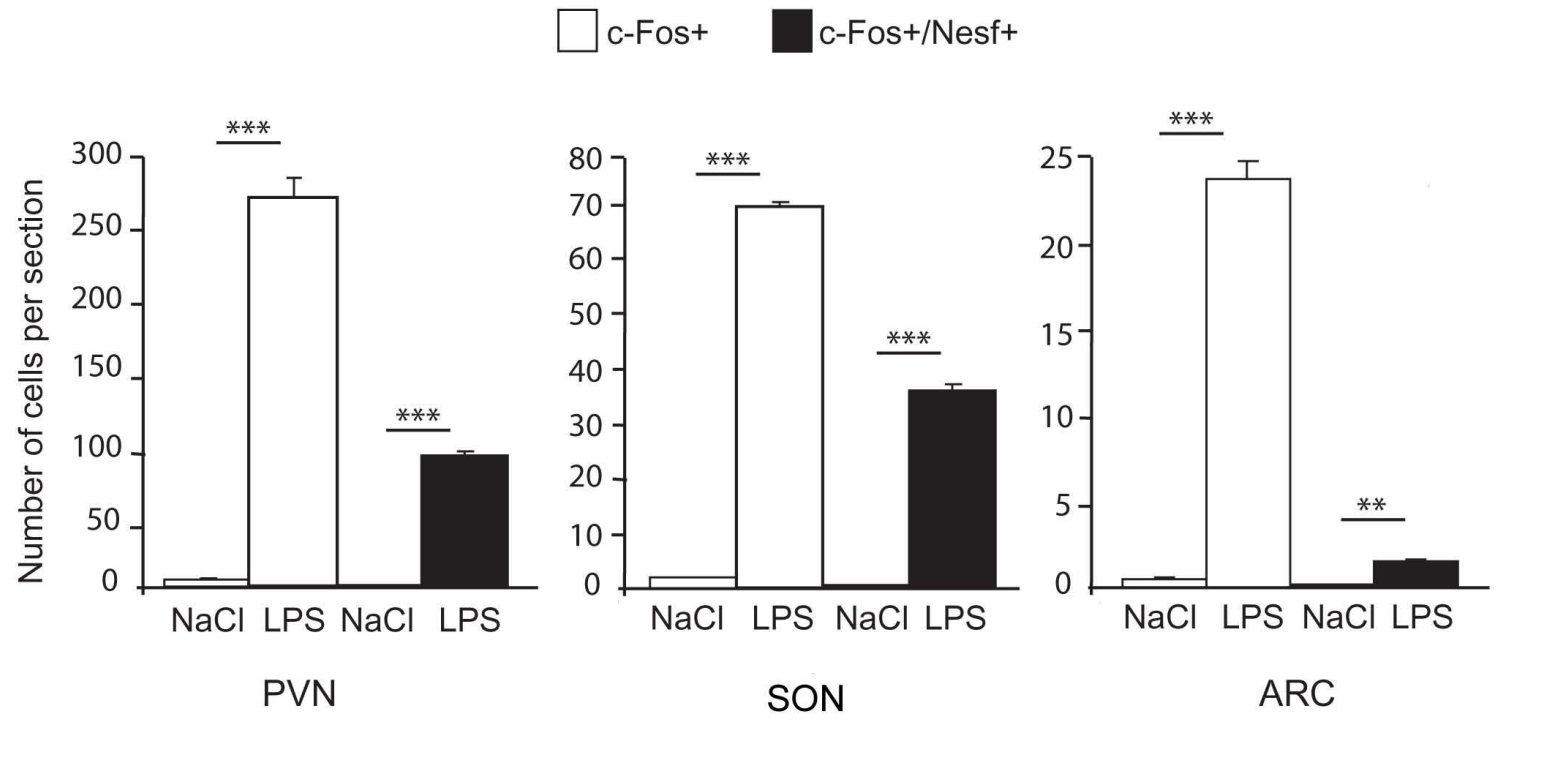
**Quantification of the number of c-Fos and c-Fos/nesfatin-1 immunoreactive cells in the PVN, SON and ARC after an i.p. injection of either saline or LPS solution**. Starred values are significantly different from saline-injected rats, ** p < 0.01; *** p < 0.001.

**Figure 4:**
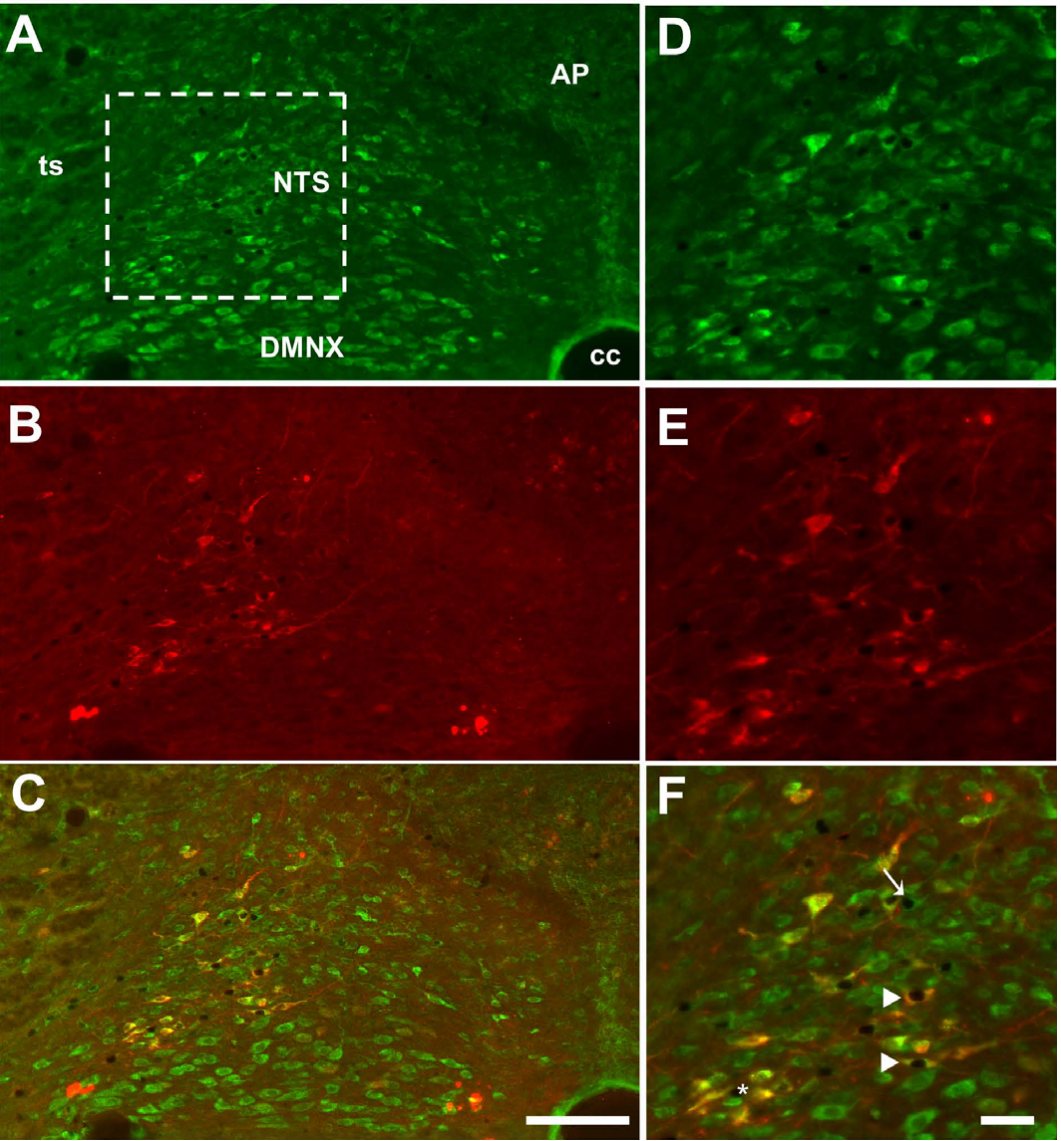
**Representative photomicrographs of c-Fos (DAB precipitate)/nesfatin-1 (green)/TH (red) triple labeling performed on brainstem coronal sections of rats treated with LPS**. Note the presence of c-Fos positive neurons co-expressing nesfatin-1 and TH (arrowheads), c-Fos+/Nesf+ neuron negative for TH (arrow) and c-Fos+/Nesf-/TH-neuron (asterisk). Dashed box in image (**A**) indicates the area where high magnification images (**D-E**) originate. AP, area postrema; cc, central canal; DMNX, dorsal motor nucleus of the vagus; NTS, nucleus of the solitary tract; ts, tractus solitarius. Scale bars: 150 μm (A-C); 40 μM (D-F).

In the hypothalamus (Figure 1A-C) and brainstem (Figure 2A) of control animals injected with physiological saline solution, c-Fos-labeled perikarya were very scarce. In contrast, rats treated with LPS exhibited an increased number of c-Fos-positive nuclei in the hypothalamus and brainstem (Figures 1D-F and 2B). This increase in c-Fos immunoreactivity was clearly detected in the PVN (261.9 ± 12.5 versus 1.3 ± 0.2 c-Fos + nuclei per section in LPS and saline-treated animals respectively; Figures 1A, D and 3), the SON (69.7 ± 0.9 versus 2.0 ± 0.1 c-Fos + nuclei per section; Figures 1B, E and 3) and the ARC (22.9 ± 0.9 versus 0.5 ± 0.1 c-Fos + nuclei per section; Figures 1C, F and 3). At the brainstem level, visualisation of c-Fos immunoreactivity revealed that LPS injection induced an increase in positive perikarya mainly within the AP and the NTS (39.7 ± 1.5 versus 0.7 ± 0.2 c-Fos + nuclei per section, Figures 2A, B and 2E). Nesfatin-1-immunoreactive neurons expressing c-Fos (c-Fos+/Nesf+) were then analyzed in hypothalamic and brainstem nuclei by means of double immunohistochemistry in both experimental groups i.e saline- and LPS-treated rats. As expected, all structures studied were virtually devoid of c-Fos+/Nesf+ neurons in control condition (Figures 2E and 3). In LPS-injected animals the greatest incidence of c-Fos/Nesf colocalization was found in SON (Figures 1H, K and 3), where 51.9 ± 2.4% of total c-Fos perikarya also express nesfatin-1. Lesser, but still highly significant, incidences were observed in the PVN and NTS where the percentage of colocalization reached 33.8 ± 2.3% and 19.71 ± 3.8%, respectively (Figure 1G, J; Figures 2 C-E and 3). Interestingly, the ARC exhibited less than 5% c-Fos/Nesf colocalization, a value (4.7 ± 0.4%) which was nonetheless significantly different from control animals (Figures 1I, L and 3). It should be noted here that despite the increased number of c-Fos nuclei in the AP of LPS-treated animals (Figure 2B), the absence of nesfatin-1 labeling in this area (Figure 2C) naturally prompted us to exclude this nucleus from our quantification. Finally, tyrosine hydroxylase (TH) immunohistochemistry revealed that within the NTS level some of the nesfatin-1 positive neurons were catecholaminergic in nature since they co-expressed TH (Figure 4A-C). Moreover, triple labeling for c-Fos/Nesf/TH indicated that about 60% of the Nesf+/TH+ neurons were activated during the immune challenge (Figure 4).

The present work confirms an hypothesized association between nesfatin-1 neurons and inflammation since we report here that some nesfatin-1-expressing neurons are activated during an inflammatory stimulus. These results therefore suggest that nesfatin-1 may intervene in the onset of physiological and behavioral changes occurring during acute phase reactions. The protocol used here, i.p. injection of LPS coupled to c-Fos staining, is classically employed to identify immunosensitive structures [3,4]. In the present study, animals received an i.p. injection of LPS at a concentration (0.25 mg/kg) reported to induce the symptoms of the acute phase reaction including fever [19], withdrawal [20] and anorexia [21] as well as c-Fos protein production in central nuclei [4,19]. Under our experimental conditions, the c-Fos distribution pattern observed throughout the brain of LPS-injected rats was consistent with the findings of previous studies performed to identify neurocircuitry involved in the coordinated physiological, endocrine, and behavioral response to LPS challenge [4,22].

The evidence of nesfatin-1-expressing neurons activation in the brainstem and hypothalamus emphasize the potential role played by these neurons in the transfer of information received from circulating immune factors. We observed that LPS injection substantially increased the number of co-labeled neurons in key nuclei activated during inflammation and involved in the coordinated physiological, endocrine and behavioral changes characteristic of sickness behavior. Nesfatin-1 has been reported to exert an anorexigenic activity when administered either centrally or peripherally [9,10]. Moreover, central nesfatin-1-expressing neurons located in the hypothalamus or brainstem are reported to be activated by refeeding or after cholecystokinine injection, which strongly suggests that these neurons partake in the complex central neurocircuitry involved in the termination of food intake [23,24]. The evidence that neurons expressing nesfatin-1, located within both the hypothalamus and brainstem, are activated during inflammation support the hypothesis that these neurons are involved in the reduction of food intake induced by inflammation. Our results imply that nesfatin-1, initially identified as a modulator of feeding behavior under physiological conditions, could also be involved in the modulation of feeding behavior during endotoxaemia anorexia. Such a duality of peptide-expressing neurons was first evoked for pro-opiomelanocortin and corticotrophin-releasing factor, hypothalamic neurons which both play an essential role in the control of energy homeostasis and are likely to be involved in inflammatory anorexia [25]. Finally, we cannot rule out the possibility that nesfatin-1 neurons activated by inflammatory signals contribute to other features of the acute-phase response. It has been proposed that the activation of neurons located in the SON contribute to a counter-hormonal response to inflammation *via *the release of neurohypophysial hormones into the circulation [26]. Interestingly, we show here that a significant fraction of LPS-activated neurons within the SON express nesfatin-1. The functional significance of such activation of nesfatin-1-expressing neurons in response to inflammation in this structure should be explored in future studies.

Double staining revealed that a large number of nesfatin-1 immunoreactive neurons of the NTS are positive for TH, indicating that a fraction of nesfatin-1 neurons belong to the A2/C2 catecholaminergic group. In accordance with the previous observation by Lacroix and Rivest [4], who reported an activation of TH-immunoreactive neurons of the NTS in response to immune challenge, triple labeling performed in the present study revealed the presence of c-Fos/nesfatin-1-positive neurons immunoreactive for TH. The activation of neurons coexpressing TH and nesfatin-1 in response to LPS might contribute to cardiovascular response to endotoxin, which consists mainly in a modification in blood pressure and heart rate [27], since catecholaminergic neurons of the NTS are involved in the regulation of cardiovascular parameters [28], and centrally injected nesfatin-1 was recently shown to affect mean arterial pressure in conscious rats [29]. The presence of c-Fos/nesfatin-1/TH triple labeled neurons in the NTS of LPS-treated rats leads also to the hypothesis that some of the nesfatin-1 neurons activated by immune challenge project to the hypothalamus. Prior research has demonstrated, for instance, the existence of catecholaminergic projections from NTS to PVN [30]. The coupling of neuronal pathway tracing studies with triple immunohistochemical labelling should be performed in the future to answer this query.

In conclusion, we show for the first time that neurons of the central nesfatinergic system are sensitive to peripheral inflammatory stimulus and thus belong to the specific immunosensitive neurocircuitry activated during infection or inflammation. Given the anorexigenic properties described for nesfatin-1 and the presence of nesfatin-1-activated neurons in key nuclei of the hypothalamus and brainstem known to contribute to food intake regulation [31], one may suggest that nesfatin-1 could account for the reduction in food intake observed during endotoxaemic anorexia. This knowledge will help better understand the neural mechanisms underlying sickness behavior.

## List of abbreviations

AP: area postrema; ARC: arcuate nucleus; DMNX: dorsal motor nucleus of the vagus; LPS: lipopolysaccharides; NTS: nucleus of the solitary tract; PVN: paraventricular hypothalamic nucleus; SON: supraoptic nucleus; TH: tyrosine hydroxylase.

## Competing interests

The authors declare that they have no competing interests.

## Authors' contributions

MD, AJ, JT and JDT designed research; MSB, EP and MD performed research; MSB, MD and JDT analyzed data and MD and JDT wrote the paper.
